# High Mortality of Wild European Rabbits during a Natural Outbreak of Rabbit Haemorrhagic Disease GI.2 Revealed by a Capture-Mark-Recapture Study

**DOI:** 10.1155/2023/3451338

**Published:** 2023-06-19

**Authors:** Saúl Jiménez-Ruiz, Marta Rafael, Joana Coelho, Henrique Pacheco, Manuel Fernandes, Paulo Célio Alves, Nuno Santos

**Affiliations:** ^1^Research Centre in Biodiversity and Genetic Resources (CIBIO), Associated Laboratory (InBIO), Campus de Vairão, University of Porto, Vairão 4485-661, Portugal; ^2^Program in Genomics, Biodiversity and Land Planning (BIOPOLIS), Campus de Vairão, Vairão 4485-661, Portugal; ^3^Animal Health and Zoonoses Research Group (GISAZ), Competitive Research Unit on Zoonoses and Emerging Diseases (ENZOEM), University of Cordoba, Cordoba 14014, Spain; ^4^Interdisciplinary Research Centre in Animal Health (CIISA), Faculty of Veterinary Medicine, University of Lisbon, Lisbon 1300-477, Portugal; ^5^Noudar Nature Park, Empresa de Desenvolvimento e Infra-Estruturas do Alqueva (EDIA), Barrancos 7230-031, Portugal; ^6^Department of Biology, Faculty of Sciences, University of Porto, Porto 4099-002, Portugal; ^7^EBM, Mértola Biological Station, Praça Luís de Camões, Mértola 7750-329, Portugal

## Abstract

Rabbit haemorrhagic disease virus (RHDV) GI.2 has caused significant declines in the abundance of wild European rabbits (*Oryctolagus cuniculus*), contributing to the species being recently classified as “endangered” in its native range. The epidemiology of this virus is still poorly understood despite its relevance for domestic and wild rabbits. During a longitudinal capture-mark-recapture (CMR) study of wild Iberian rabbits, *O. c. algirus*, in a semiextensive breeding enclosure, an outbreak of RHDV GI.2 took place in January-February 2022, allowing us to estimate key epidemiological parameters of a natural outbreak. From April 2021 to July 2022, 340 rabbits were captured 466 times and individually identified, and some were vaccinated against myxoma virus (MYXV) and/or RHDV GI.2. Sera were collected and tested for IgG specific for MYXV and RHDV GI.2, and data were analyzed using multievent CMR models. During six weeks in January-February 2022, an estimated 81.0% (CI_95_ 77.1–84.3%) of the population died. Intensive aboveground searches could recover 189 carcasses (50.5% of the estimated mortality, CI_95_ 41.8–63.4%), with RHDV GI.2 detected in 6/7 tested. Apparent RHDV GI.2 seroprevalence rose from 15.4% (CI_95_ 8.0–27.5%) in January 2022 to 87.9% (CI_95_ 72.7–95.2%) in February 2022. The apparent mortality of RHDV GI.2-seropositive rabbits during the outbreak was estimated as null, while for seronegative rabbits, it was 76.0% (CI_95_ 53.8–90.3%). Among the seronegative rabbits, mortality was higher in unvaccinated (100%) than in recently vaccinated (60.0 ± 16.6%) and in females (100%) than in males (52.0 ± 17.1%). Infected carcasses in the burrows might explain the medium-term disease persistence in the population following the outbreak. Rabbits with antibodies at the cutoff for seropositivity were fully protected from fatal infection. Females had a higher fatality rate than males, underscoring the impact of RHDV GI.2 on the population dynamics of this endangered species.

## 1. Introduction

Capture-mark-recapture (CMR) studies are based on the capture of animals using invasive (e.g., trapping) or noninvasive methods (e.g., faecal samples) and their individual identification using natural (e.g., phenotype or genotype) or artificial marks (e.g., tags). The animals are subsequently released and eventually recaptured [1]. Such studies were initially developed to understand ecological processes in wildlife, including population demography, recruitment, and survival rates [2, 3]. This approach was then extended to epidemiological studies [4]. Inferences from CMR studies have contributed to the management of wild species and their pathogens [5, 6].

European rabbits (*Oryctolagus cuniculus*) were highly abundant in their native range in the Iberian Peninsula and Southern France until the introduction of the myxoma virus (MYXV) in the 1950s [7]. The European rabbit is a keystone species in its native range as it is a fundamental part of the diet of many predators, ecosystem engineers, and seed dispersers providing habitat for other species [8]. Rabbit populations have undergone a decades-long decline culminating in the recent reclassification by the International Union for Conservation of Nature as “endangered” in its native range [9]. Pathogens, particularly MYXV and rabbit haemorrhagic disease virus (RHDV), are significant drivers of this decline [10–13].

Rabbit haemorrhagic disease virus is a Lagovirus that causes fatal haemorrhagic hepatitis in domestic and wild European rabbits, whose GI.1 variant has been present in the Iberian Peninsula since 1988 [11]. A new variant RHDV GI.2, first detected in France in 2010 [14], rapidly spread throughout Europe, replacing the previous GI.1 strain [15–17]. The GI.2 variant is antigenically and epidemiologically distinct from the GI.1, notably showing high pathogenicity in juvenile rabbits [18–20]. The RHDV GI.2 caused significant declines in European rabbit abundance following its emergence in the Iberian Peninsula and elsewhere [21, 22]. While the pathogenicity of RHDV GI.2 in European rabbits was thoroughly studied through experimental infections [19, 23], its epidemiology in wild populations in its native range is still largely unknown, particularly for the southwestern Iberian subspecies (*Oryctolagus cuniculus algirus*).

A comprehensive understanding of the epidemiological processes that influence pathogen dynamics in host populations is critical for preventing and controlling diseases [24]. Capture-mark-recapture studies could provide information on the epidemiology of RHDV GI.2 in wild European rabbit populations and offer potential applications in disease control [25]. The demography and epidemiology of a population of the southwestern Iberian subspecies of the European rabbit have been studied since 2021 through a longitudinal CMR. The present study aims to describe key epidemiological parameters of an outbreak of RHDV GI.2 during January and February 2022, providing useful information for disease control in wild and domestic populations.

## 2. Materials and Methods

### 2.1. Study Area

A longitudinal CMR study of European rabbits was performed in a 2.3-hectare semiextensive breeding enclosure at Parque Natureza Noudar (PNN), a 1,000 ha private estate in southeastern Portugal (38°11′03.7″N, 7°02′24.4″W). The landscape at the breeding enclosure and surroundings consist of a mosaic of scrub (mostly *Cistus* sp.) sparsely forested with holm oak (*Quercus ilex*). A perimetral 2 m-high rabbit-proof fence with electrical wire prevented access by terrestrial carnivores and rabbit emigration. Water and commercial feed were provided *ad libitum *year-round in the enclosure. Our team is performing a long-term longitudinal study at several sites in Portugal; the results obtained from 2019 to 2020 at another enclosure in the same estate were previously reported [26].

### 2.2. Study Design and Sampling

**Figure 1:**
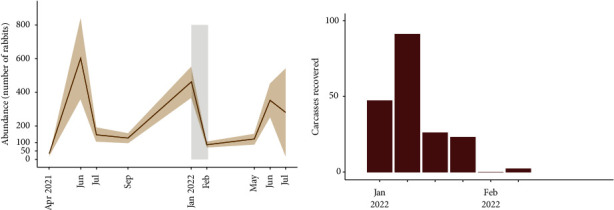
Rabbit abundance and carcasses recovered during the outbreak. (a) Estimated abundance of rabbits in each trapping session with standard error. Trapping sessions in the *X*-axis, light grey rectangle highlights the rabbit haemorrhagic disease virus GI.2 outbreak duration. (b) Weekly number of rabbit carcasses recovered during the outbreak.

Fifteen cage traps were permanently placed near the pasture and feeding troughs in the enclosure. They were set 2 hours before sunset, baited with vegetables, checked 2 hours after sunset, and again 1 hour after sunrise. Cage traps were closed during the day. Between April 2021 and July 2022, nine trapping sessions of two or three occasions (nights) each were performed in April, June, July, and September 2021, and January, February, May, June, and July 2022 (see *X*-axis in Figure 1), in which 340 rabbits were captured 466 times.

Each trapped rabbit was individually identified with a subcutaneous microchip when first captured. A volume of 0.5–1.5 ml of whole blood (<0.25% body weight) was collected by venipuncture of the saphenous vein, placed into sterile clotting tubes, and centrifuged, and sera were collected and stored at −20°C until analysis. Blood samples were collected whenever the previous sampling of the individual rabbit had occurred >1 month before. Rabbits were released at the capture site, except for 200 rabbits translocated to restock other breeding enclosures or free-ranging populations within PNN.

Seventy-nine of the 146 rabbits released in the same enclosure were vaccinated with a homologous inactivated RHDV GI.2 vaccine (ERAVAC®, HIPRA, Spain), and 61 of those 79 were also vaccinated with a heterologous live MYXV vaccine employing Shope fibroma virus (MIXOHIPRA-FSA®, HIPRA, Spain), using the subcutaneous dosages recommended by the manufacturers (0.5 ml). Until January 2022, before the observed epidemic outbreak of RHDV, only 36 RHDV GI.2-vaccinated rabbits were released back in the same enclosure; 33 of those 36 were also vaccinated for MYXV.

To prevent pathogen transmission between individuals and study sites, rabbits were kept in individual cleaned cloth bags and handled with latex gloves over disposable pads, and the equipment was sanitized with disinfectant wipes after each trapping occasion. Live trapping and sample collection were conducted under permits 23/2021 and 574/2022, according to European Union directives on the protection of animals used for scientific purposes (Directive 2010/63/EU) and international wildlife standards [27, 28].

### 2.3. Rabbit Haemorrhagic Disease Virus Outbreak

Five freshly dead rabbits were found in the breeding enclosure at PNN during the final trapping occasion in the January 2022 session (January 20^th^). After that, rabbit carcasses were thoroughly searched aboveground in the enclosure two or three times a week. Carcasses were collected under biosecurity measures, buried outside the enclosure and covered in potassium hydroxide. Efforts to locate carcasses inside burrows were not taken to minimize the risk of disturbing rabbit litters during the peak birth season. A subset of the recovered carcasses (*n* = 7, including the five initial ones) were frozen until necropsy. Liver samples were collected and tested for RHDV GI.2 using a chromatographic lateral flow assay (INGEZIM® RHDV1/2 DIF CROM, EUROFINS, Spain). This assay for RHDV GI.1 and GI.2 was recently validated in liver samples of European rabbits, showing a sensitivity of 94.4% and specificity of 100% compared to molecular methods [29]. RHDV GI.2 was detected in 6 out of the 7 necropsied rabbits.

### 2.4. Serological Analyses

An in-house indirect ELISA (iELISA) to detect IgG specific against RHDV GI.2 [30] was performed with minor modifications, as previously described by Pacheco et al. [26]. Briefly, GI.2-derived virus-like particles (VLP), expressed in a baculovirus expression system and purified according to Almanza et al. [31], were diluted in carbonate/bicarbonate buffer (pH = 9.5) and absorbed to Nunc Maxisorp 96 wells ELISA plates (100 ng/well) by overnight incubation at 4°C. The plates were blocked with phosphate-buffered saline (PBS)-5% skim milk solution and washed three times. Sera samples were tested at a 1/200 dilution in PBS-5% skim milk solution. The conjugate goat anti-rabbit IgG/HRP (Bio-Rad) was added at 1/4,000 dilution, followed by the substrate (3.3′, 5.5′-tetramethylbenzidine) (Abcam), reactions stopped with 100 *µ*l of 1 M phosphoric acid, and the optical density at 450 nm (OD_450 nm_) recorded within 15 min. Positive controls consisted of pooled sera of rabbits with high iELISA readings [26]. Negative controls were pooled sera of unvaccinated domestic European rabbits raised indoors without a history of clinical disease. To detect specific IgG against MYXV, a commercial iELISA kit (INGEZIM® 17.MIX.K1, EUROFINS, Spain) was performed according to the manufacturer's instructions.

All serum samples, positive and negative controls, were tested in duplicate. The tests were valid if the average OD_450 nm_ of the two replicates of the positive control was >5x the average OD_450 nm_ of the two replicates of the negative control. The iELISA results were standardized by the normalized absorption ratios (NAR) according to the following equation:

(1) $$\begin{array}{c} NAR=\frac{average\;OD450nm\;sample}{2\;x\;\left(average\;OD450nm\;negative\;control\right)}\text{.} \end{array}$$

The cutoff for seropositivity was set at NAR = 2.0 for RHDV GI.2 and NAR = 2.4 for MYXV, as previously described [26]. These tests using these cutoffs achieved 100% diagnostic sensitivity and specificity [26].

### 2.5. Multievent Capture-Mark-Recapture Models

Multievent CMR (MECMR) models were applied to serological data for each trapping session [32]. A single-state goodness-of-fit test was performed in U-CARE [33]. Test 3.SR indicated significant transience (*χ*^2^ = 3.814, *P* < 0.001, 7 df). Transience can be defined as individuals captured for the first time having a lower probability of being recaptured when compared with individuals previously captured [34]. No trap dependence was identified (test 2.CT *χ*^2^ = −1.411, *P*=0.158, 6 df).

Models were implemented using the software E-SURGE [35]. The model included the matrices: initial state, survival (time-varying survival probability), transitions between seropositive and seronegative states (time-varying seroconversion probability; constant seroreversion probability), detection (time-varying capture probability), probability of being tested, and uncertainty in state assignment (corresponding to the diagnostic sensitivity and specificity; see [26]) (Supplementary material, Appendix S1). A quasi-Newton nonlinear solver was used to obtain the maximum likelihood estimator, and 50 models run using different sets of random initial values were applied to avoid local minima.

The following nonobservable states were considered in the models: seronegative (S−), seropositive (S+), and dead (D). In any sampling session, an individual rabbit may be alive in classes S−, S+, or may be dead. In each sampling session, the possible observations were as follows: not detected (0), detected S− (1), detected S+ (2), or detected but not tested (3). The probability of being assigned the event “detected but not tested” was fixed as the proportion of detections where no blood was collected and no iELISA result was obtained (38.3% of the captures). Models were selected under an information-theoretical approach by their Akaike information criterion corrected for small sample size (AICc) [36]. Unequal time intervals between sampling occasions were defined, with an interval 1 corresponding to 30 days, meaning the estimated parameters are monthly probabilities. The rabbits removed from the study population were right-censored, so they did not contribute to the demographic or epidemiological parameters after their last capture.

Rabbit abundance was estimated for each trapping session using nonspatial Jolly–Seber–Schwarz–Arnason (JSSA) CMR models. They were implemented using the package “openCR” v. 2.2.4 [37] in R 3.6.1 [38] through the interface Rstudio 2022.07.1 [39].

## 3. Results

### 3.1. During the Outbreak

The rabbit population in the enclosure was estimated at 462 ± 93 (mean ± standard error) rabbits on January 18–20^th^, while it was 88 ± 17 on February 22–24^th^ (Figure 1(a)). Assuming a closed population, the total mortality during the first five weeks of the outbreak was estimated at 374 rabbits (CI_95_ 298–452), corresponding to 81.0% (CI_95_ 77.1–84.3%; 374/462) of the population before the outbreak. From January 20^th^ to March 2^nd^, 189 rabbit carcasses were collected aboveground, most of them (73.0%) in the first two weeks of the outbreak (Figure 1(b)). The recovered carcasses correspond to 50.5% (CI_95_ 41.8–63.4%, 189/374) of the estimated total mortality during the first five weeks of the outbreak.

**Figure 2:**
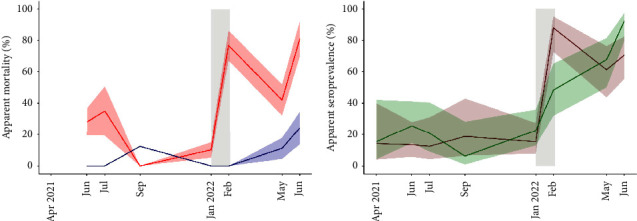
Apparent mortality and seroprevalence during the study. (a) Monthly apparent mortality of rabbits seropositive (blue) and seronegative (red) for rabbit haemorrhagic disease virus (RHDV GI.2). (b) Apparent seroprevalence of RHDV GI.2 (dark orange) and myxoma virus (green) with 95% confidence intervals. Trapping sessions in the *X*-axis, light grey rectangle highlights the rabbit haemorrhagic disease virus GI.2 outbreak duration.

Apparent mortality during the January-February 2022 outbreak peaked at 76.7% (CI_95_ 53.8–90.3%) for RHDV-seronegative rabbits, while it was estimated as null for the seropositive ones (Table 1 and Figure 2(a)). A previous minor peak in the apparent mortality of RHDV-seronegative rabbits occurred in June-July 2021 (35.0%, CI_95_ 12.2–67.5%), without carcasses being detected (Figure 2(a)). The apparent mortality of RHDV-seropositive rabbits rose slightly in June 2022 (Figure 2(a)).

From April 2021 to January 2022, the apparent seroprevalence of RHDV GI.2 was stable and low, ranging from 12.5% (CI_95_ 4.3–31%) to 15.4% (CI_95_ 8.0–27.5%). In February 2022, the RHDV seroprevalence sharply increased to 87.9% (CI_95_ 72.7–95.2%), remaining high until June of the same year (Figure 2(b)). The same pattern was observed for MYXV, except that seroprevalence rose gradually from 22.5% (CI_95_ 13.0–35.9%) in January 2022 to 92.3% (CI_95_ 79.7–97.4%) in June 2022 (Figure 2(b)).

**Figure 3:**
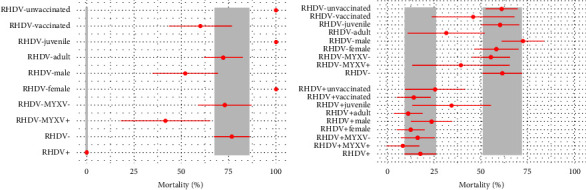
Determinants of apparent monthly mortality during and after the outbreak. Effects of the serological status for myxoma and rabbit haemorrhagic disease virus (RHDV GI.2), sex, age, and prior vaccination on the apparent monthly mortality, with standard errors. (a) During the outbreak: parameters estimated from January 20^th^ to February 24^th^, 2022. (b) Postoutbreak: parameters estimated from February 24^th^ to June 16^th^, 2022. Light grey bars highlight the apparent mortality of RHDV GI.2-seropositive and RHDV GI.2-seronegative rabbits.

The most supported MECMR model included the effect of vaccination during the January 2022 trapping session on rabbit survival during the RHDV GI.2 outbreak (Model 1, Table S1). Vaccination at the start of the outbreak decreased the mortality of previously seronegative rabbits from 100% to 60.0% ± 16.6% (Table 1 and Figure 3(a)). The monthly probability of seroreversion was estimated as null (Model 1, Table S1).

The models including the effect on rabbit survival during the outbreak of the serological status for MYXV (ΔAICc = 2.22, Model 2, Table S1) and sex (ΔAICc = 2.25, Model 3, Table S1) were almost equally supported. Rabbits seronegative for both RHDV GI.2 and MYXV showed higher apparent mortality (72.9% ± 14.2%) than those that were seronegative to RHDV GI.2 but seropositive to MYXV (41.5% ± 23.5%) (Table 1 and Figure 3(a)). Among the RHDV GI.2-seronegative rabbits, females showed higher mortality (100% ± 0%) than males (52.0% ± 17.1%) (Table 1 and Figure 3(a)).

The model that included the effect of age on rabbit survival during the outbreak was weakly supported (ΔAICc = 4.77, Model 5, Table S1) and estimated higher mortality in juveniles, both RHDV GI.2-seronegative (100% ± 0% vs. 72.1% ± 10.2% for adults) and seropositive (50.0% vs null for adults) (Table 1 and Figure 3(a)).

### 3.2. Postoutbreak

The same MECMR analysis was performed for May–June 2022 to assess the postoutbreak epidemiological scenario. After the January-February outbreak, the apparent mortality increased in RHDV GI.2-seropositive rabbits, and it decreased in RHDV GI.2-seronegative ones (Figure 3(b)). The mortality of RHDV GI.2-seropositive/MYXV-seronegative rabbits rose from 0% during the outbreak to 16.2% ± 9.1% postoutbreak (Figure 3(b)).

### 3.3. Minimum Protective Humoral Immunity

**Figure 4:**
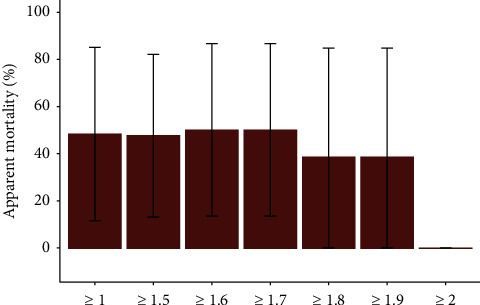
Apparent mortality of seropositive rabbits across a range of thresholds for seropositivity. Estimated apparent mortality from January 20 to February 24, 2022, during the rabbit haemorrhagic disease virus GI.2 outbreak, across a range of normalized absorbance ratios cutoff thresholds for seropositivity.

The most supported model (Model 1, Table S1) was further explored to assess the minimum level of humoral immunity that fully protects rabbits from fatal RHDV GI.2 infection. The apparent mortality of RHDV GI.2-seropositive rabbits in January-February 2022 was estimated across a range of cutoff thresholds for seropositivity (Figure 4). For rabbits with iELISA normalized absorbance ratios slightly below the diagnostic threshold (NAR ≥1.9), it showed increased mortality during the outbreak, compared to the null mortality estimated for those at or above that threshold (NAR ≥2.0).

## 4. Discussion

An epidemic of RHDV GI.2 in a European rabbit population undergoing a detailed longitudinal study allowed us to estimate some critical epidemiological parameters of this pathogen of significant relevance for the conservation of this keystone species [8, 12, 13]. While the epidemiology of RHDV GI.1 in wild populations was studied soon after its emergence (e.g., [40, 41]), that of RHDV GI.2 has only been addressed through experimental infections (e.g., [18, 23, 42, 43]).

The reported case fatality of RHDV GI.2 was variable [14, 42], with higher values associated with recent strains, suggesting viral evolution towards higher pathogenicity [18]. Our results support the high pathogenicity of the strains circulating in the Iberian Peninsula based on the overall mortality of seronegative rabbits (76.7 ± 9.5%) over five weeks of the outbreak (Table 1). Although our study does not allow us to estimate the case fatality of RHDV GI.2 directly, the large scale of the outbreak suggests it was extremely high. Given an estimated rabbit abundance of 88 ± 17 and seroprevalence of 87.9% (CI_95_ 72.7–95.2%) after the outbreak, only 11 rabbits (CI_95_ 9–13) were not infected. In such a scenario, where the timeframe of the outbreak was short, almost all individuals were infected, and emigration is impossible, the apparent mortality should be a close proxy of the case fatality [44].

The RHDV GI.2 outbreak occurred during the breeding season, between January and February, according to the phenology described in wild populations of European rabbits [43, 45]. Declines of 60–80% in the rabbit populations over several years have been reported following the emergence of RHDV GI.2 in naïve rabbit populations in the Iberian Peninsula and Australia [21, 22]. Such drastic declines can unbalance ecosystems, e.g., by reducing the fecundity of rabbit predators [22]. 14 hours study documented a reduction of 81% in rabbit abundance after a large outbreak. Abundance increased in the following spring (353 ± 99 rabbits in July 2022), although it did not attain the values of the previous year (Figure 1(a)). It should be noted that the density of the study population was unnaturally high (>200 rabbits/hectare) as the apparent mortality was <20% before the outbreak, as expected for an enclosure with supplemental feeding and no terrestrial predators [46]. The seroprevalence before the outbreak was extremely low (12.5%–15.6%), which suggests that our model system could be close to a naïve population [47].

The detected mortality spanned six weeks, concentrated in the first two weeks of the outbreak (Figure 1(b)). Only approximately half of the estimated number of dead rabbits could be recovered despite intensive aboveground searches for carcasses. Given the perimetral rabbit- and carnivore-proof fence, two hypotheses can explain this observation: first, some of the carcasses could have been removed and consumed by avian scavengers, which are abundant in this estate because of the conservation-oriented management; second, it could be due to many rabbits dying inside the burrows, thus contributing to the persistence of RHDV GI.2 [20]. This hypothesis was supported by the evidence of the continuing circulation of RHDV GI.2 in the months following the detected outbreak, as seronegative rabbits continued to exhibit higher mortality than seropositive ones (Figures 2(a) and 3(b)).

We found evidence of the prolonged circulation of MYXV, starting in January 2022, when MYXV seroprevalence rose from very low levels (6.3%), achieving 92.3% in June 2022 (Figure 1(b)). Furthermore, the mortality of RHDV GI.2-seropositive/MYXV-seronegative rabbits rose from 0% in January-February to 17.7 ± 8.6% in May–June (Figure 3), supporting the circulation of MYXV in the postoutbreak period. During the outbreak, the mortality of RHDV-seronegative MYXV-seropositive rabbits (41.5% ± 23.5%) was lower than that of RHDV-seronegative MYXV-seronegative rabbits (72.9% ± 14.2%). While this could suggest the co-circulation of both viruses, RHDV-seropositive rabbits showed no difference in mortality according to the serological status for MYXV (0% for both MYXV-seropositive and negative). Together, these results support that humoral immunity to MYXV might reduce RHDV GI.2 pathogenicity, in contrast to what was suggested for RHDV GI.1 in Australia [48]. Unspecified differences in the immune response to RHDV GI.1 and GI.2 variants [49, 50] might explain these differences, was as well as the complex interactions between other pathogens and the immune response to MYXV and RHDV GI.2 [42, 51].

The apparent seroprevalence of RHDV GI.2 was stable and low (12.5–15.4%) until January 2022, despite the vaccination against RHDV GI.2 of 36 rabbits released back into this enclosure, which suggests that the virus was not circulating before that date. A slight peak in the mortality of RHDV-seronegative rabbits (35.0%; Figure 2(a)) occurred in June-July 2021 without detected mortality. This observation suggests a minor outbreak of RHDV in the summer of 2021, without the persistence of viral circulation in the following months.

Our study evidenced two relevant parameters related to the immunology of RHDV GI.2. First, the probability of seroreversion (shifting from seropositive to seronegative) was estimated as null by MECRM models. These results support the presence of lifelong immunity against this agent. Second, despite the high case fatality of RHDV GI.2 infections, the humoral immunity developed against this virus seems to be highly protective because the mortality of seropositive rabbits was also estimated as null during the outbreak (Table 1 and Figure 2(a)). Furthermore, our results suggest that the minimum level of humoral immunity that fully protects from fatal infection coincides with the iELISA cutoff threshold for seropositivity of NAR = 2 (Figure 4). Strikingly, even a slightly lower NAR ≥1.9 yields an estimate of 38.4% mortality in seropositive rabbits, compared to 0% in rabbits with NAR ≥2.0. Single-dose subcutaneous vaccination with commercial inactivated RHDV GI.2 virus right before the outbreak decreased the mortality of previously seronegative rabbits from 100% to 60 ± 16.6%. This observation suggests that vaccination at the outbreak's start does not fully protect from fatal infection, despite attenuating its impact.

The MECMR models also showed higher mortality during the January-February outbreak in female RHDV GI.2-seronegative rabbits (100%) compared to males (52.0 ± 17.1%) (Table 1 and Figure 3). Experimental infections did not report differences in the susceptibility to RHDV GI.2 between sexes [18, 19, 23, 42, 50, 52, 53], but it is unclear whether this potential risk factor was included in some of the analyses. Our data underscore the demographic impact of RHDV GI.2 in wild European rabbits, highlighting the effect of a single outbreak in drastically reducing the number of breeding females in a population at the peak of the breeding season, which could significantly impair its recovery. Interestingly, in the postoutbreak period where RHDV GI.2 and MYXV cocirculated, mortality in males was slightly higher than in females, irrespective of the serological status for RHDV GI.2 (Figure 3).

One of the most notable characteristics of RHDV GI.2 is its high pathogenicity in juvenile rabbits, with case fatality upon experimental infection estimated at 21–100% [18, 19, 23, 42, 50, 52, 53] and in adult rabbits at 0–89% [19, 23, 52]. The MECMR model including the effect of age was weakly supported but suggested higher mortality in juvenile rabbits, either seropositive (50% vs. 0% in adults) or seronegative (100% vs. 72.1 ± 10.2% in adults) to RHDV GI.2 (Table 1).

The most notable aspect of the field epidemiology of RHDV GI.2 is the high pathogenicity in juveniles, even if seropositive (Table 1), confirming the results of experimental infections [19, 42]. By contrast, RHDV GI.1 was weakly pathogenic for this age class [40]. Another relevant aspect of RHDV GI.2 is the complete protection from fatal infection provided by circulating antibodies (Table 1), in contrast to the significant mortality induced by RHDV GI.1 in seropositive rabbits [41, 54].

This study supports the high case fatality rate of RHDV GI.2 in seronegative European rabbits during natural outbreaks and the effective protection conferred by humoral immunity. The initial abundance in the enclosure was extremely high, and despite a low seroprevalence (15.4%), the number of survivors was large enough for the prompt recovery of the population. This study highlights the threat posed by RHDV GI.2 to isolated low-abundance populations of European rabbits and other threatened lagomorph taxa where this pathogen recently emerged, such as the riparian brush rabbit (*Sylvilagus bachmani riparius*) and pygmy rabbit (*Brachylagus idahoensis*) in North America [55, 56]. Populations with high abundance and/or seroprevalence should be a management goal for conserving endangered lagomorphs in their native ranges.

## 5. Conclusions

The detailed longitudinal demographic and epidemiological study allowed us to estimate some key epidemiological parameters of a natural epidemic of RHDV GI.2 in a population of European rabbits. We estimated the apparent mortality during and postoutbreak, highlighting the overall high pathogenicity and demographic impact of circulating RHDV GI.2 strains. The detectable epidemic had a short course of six weeks. Still, the virus apparently kept circulating in the following months, probably facilitated by the environmental contamination arising from a proportion of infected carcasses remaining inside burrows. Specific IgG is fully protective against fatal infection by RHDV GI.2 at the seropositivity threshold, but subsequent MYXV infection might negatively impact the immune response. Vaccination at the outbreak's start is only partially protective against fatal disease. The novel information provided by this study contributes to a better understanding of the epidemiology of RHDV GI.2, a pathogen of major relevance for conserving endangered keystone species.

## Figures and Tables

**Table 1:** Monthly apparent mortality during the outbreak of rabbit haemorrhagic disease virus GI.2. Estimated mortality between January 20^th^ and February 24^th^, 2022, from the models detailed in Table S1.

Variables	Classes	RHDV GI.2 seropositive	RHDV GI.2 seronegative	Model
		Mean ± standard error (%)	Mean ± standard error (%)	
n. a.	All rabbits	0 ± 0	76.7 ± 9.5	Model 4

Vaccine	Vaccinated^(1)^	0 ± 0	60.0 ± 16.6	Model 1
	Unvaccinated	0 ± 0	100 ± 0	

Myxoma virus (MYXV)	Seropositive to MYXV	0 ± 0	41.5 ± 23.5	Model 2
	Seronegative to MYXV	0 ± 0	72.9 ± 14.2	

Sex	Females	0 ± 0	100 ± 0	Model 3
	Males	0 ± 0	52.0 ± 17.1	

Age	Adults	0 ± 0	72.1 ± 10.2	Model 5
	Juveniles	50.0 ± 0	100 ± 0	

n. a., not applicable.^(1)^Vaccinated during the January 2022 trapping session.

## Data Availability

All data supporting the findings of the present study are available upon reasonable request from the corresponding author.

## Supplementary Materials

Supplementary Materials Appendix S.1: summary of the methodological approach for the multievent capture-mark-recapture models. Table S1: summary of the model selection.
