# Supplementary material for: High Mortality of Wild European Rabbits during a Natural Outbreak of Rabbit Haemorrhagic Disease GI.2 Revealed by a Capture-Mark-Recapture Study
Source: Transbound Emerg Dis. 2023 Jun 19;2023:3451338. doi: 10.1155/2023/3451338 (PMC12017207; doi:10.1155/2023/3451338)
Supplement: Supplementary Materials — Appendix S.1: summary of the methodological approach for the multievent capture-mark-recapture models. Table S1: summary of the model selection. [file 3451338.f1.docx]

**Supplementary material**

**High mortality of wild European rabbits during a natural outbreak of rabbit haemorrhagic disease GI.2 revealed by a capture-mark-recapture study**

**Saúl Jiménez-Ruiz^1,2,3^, Marta Rafael^1,2^, Joana Coelho^1,4^, Henrique Pacheco^1,4^, Manuel Fernandes^5^, Paulo Célio Alves^1,2,6,7^ Nuno Santos^1,2,7^**

^1^ Centro de Investigação em Biodiversidade e Recursos Genéticos (CIBIO), Laboratório Associado (InBIO), Campus de Vairão, Universidade do Porto, 4485-661 Vairão, Portugal

^2^ Program in Genomics, Biodiversity and Land Planning (BIOPOLIS), Campus de Vairão, 4485-661 Vairão, Portugal

^3^ Animal Health and Zoonoses Research Group (GISAZ), Competitive Research Unit on Zoonoses and Emerging Diseases (ENZOEM), University of Cordoba, 14014 Cordoba, Spain

^4^ Centro de Investigação Interdisciplinar em Sanidade Animal (CIISA), Faculty of Veterinary Medicine, University of Lisbon, 1300-477 Lisbon, Portugal

^5^ Parque de Natureza de Noudar, Empresa de Desenvolvimento e Infra-estruturas do Alqueva (EDIA), 7230-031 Barrancos, Portugal

^6^ Departamento de Biologia, Faculdade de Ciências, Universidade do Porto, 4099-002 Porto, Portugal

^7^ EBM, Estação Biológica de Mértola, Praça Luís de Camões, 7750-329 Mértola, Portugal

**Appendix S.1. Summary of the methodological approach for the multi-event capture-mark-recapture models.**

**Table S1. Summary of the model selection.**

**Appendix S.1.** **Summary of the methodological approach for the multi-event capture-mark-recapture models**

Assuming the initial state vector *IS*, the survival matrix *S*, the transition matrixes *T_serology_* and *T_awareness_*, the test matrix *M* and the event matrix *B* (Pradel, 2005; Pradel & Sanz-Aguilar, 2012). If π denotes the proportion of newly marked individuals in the state A+ and 1- π in state A-, φ the probability that an individual survives from *t* to *t+1*, ψ the probability that it changes serological state, *p* the probability that it is captured, *m* the probability that an individual sample is tested, and *b* the probability that it is assigned a given observation (event):

|  | A+ | A- | D |
| --- | --- | --- | --- |
| *IS* = | π | 1-π | 0 |

Gemaco syntax: to

|  |  | A+ | A- | D |
| --- | --- | --- | --- | --- |
|  | A+ | Φ | 0 | 1- Φ |
| S = | A- | 0 | Φ | 1- Φ |
|  | D | 0 | 0 | 1 |

Gemaco syntax: f.t+t(5).cov

|  |  | A+ | A- | D |
| --- | --- | --- | --- | --- |
|  | A+ | 1- ψ | ψ | 0 |
| Tserology = | A- | ψ | 1- ψ | 0 |
|  | D | 0 | 0 | 1 |

Gemaco syntax: f(1).to(2)+f(2).to(1).t

|  |  | ND | A+ | A- |
| --- | --- | --- | --- | --- |
|  | A+ | 1-*p* | *p* | 0 |
| *D* = | A- | 1-*p* | 0 | p |
|  | D | 1 | 0 | 0 |

Gemaco syntax: t

|  |  | ND | A+ tested | A- tested | Not tested |
| --- | --- | --- | --- | --- | --- |
|  | ND | 1 | 0 | 0 | 0 |
| *M* = | A+ | 0 | 1-*m* | 0 | *m* |
|  | A- | 0 | 0 | 1- *m* | *m* |

Gemaco syntax: i

|  |  | 0 | 1 | 2 | 3 |
| --- | --- | --- | --- | --- | --- |
|  | ND | 1 | 0 | 0 | 0 |
|  | A+ tested | 0 | 1-*b* | *b* | 0 |
| *B* = | A- tested | 0 | *b* | 1-*b* | 0 |
|  | Not tested | 0 | 0 | 0 | 1 |

Gemaco syntax: f

The possible events were not detected (0), detected seronegative (1), detected seropositive (2), detected not tested (3). The probability of encounters not being tested (*m*) was fixed as the proportion of detections (captures) where no blood samples were collected (0.383). The covariates in the survival probability at time 5 were sex, age (adult, juvenile), and serological status for MYXV (seronegative/seropositive).

**Table S1. Summary of the model selection.** Models with ΔAICc < 5 and the model correcting for transience are shown. Rabbit haemorrhagic disease (RHDV GI.2) outbreak from January, 20^th^ to February 24^th^, 2022.

| **RHDV** **GI.2** | **Model parametrization** | | | | | **nP** | **Deviance** | **AICc** | **ΔAICc** |
| --- | --- | --- | --- | --- | --- | --- | --- | --- | --- |
|  | **Survival**  (*S*) | **Serology**  (*T_serology_*) | **Detection**  (*D*) | **Testing**  (*M*) | **State assignment**  (*B*) |  |  |  |  |
| Model 1 | Time effect  Prior vaccination effect during RHDV GI.2 outbreak | Time effect on seroconversion  Constant seroreversion | Time effect on detection  Transience not accounted | Probability not being tested fixed (0.383) | Constant specificity  Sensitivity fixed (1.0) | 35 | 1137.31 | 1213.92 | 0 |
| Model 2 | Time effect  **MYXV effect during RHDV** **GI.2 outbreak** | Time effect on seroconversion  Constant seroreversion | Time effect on detection  Transience not accounted | Probability not being tested fixed (0.383) | Constant specificity  Sensitivity fixed (1.0) | 36 | 1137.17 | 1216.14 | 2.22 |
| Model 3 | Time effect  **Sex effect during RHDV GI.2 outbreak** | Time effect on seroconversion  Constant seroreversion | Time effect on detection  Transience not accounted | Probability not being tested fixed (0.383) | Constant specificity  Sensitivity fixed (1.0) | 36 | 1137.29 | 1216.17 | 2.25 |
| Model 4 | **Time effect** | Time effect on seroconversion  Constant seroreversion | Time effect on detection  Transience not accounted | Probability not being tested fixed (0.383) | Constant specificity  Sensitivity fixed (1.0) | 34 | 1143.27 | 1217.39 | 3.47 |
| Model 5 | Time effect  **Age effect during RHDV** **GI.2 outbreak** | Time effect on seroconversion  Constant seroreversion | Time effect on detection  Transience not accounted | Probability not being tested fixed (0.383) | Constant specificity  Sensitivity fixed (1.0) | 36 | 1139.70 | 1218.69 | 4.77 |
| Model 6 | Time effect  Prior vaccination effect during RHDV GI.2 outbreak | Time effect on seroconversion  Constant seroreversion | Time effect on detection  **Transience accounted** | Probability not being tested fixed (0.383) | Constant specificity  Sensitivity fixed (1.0) | 34 | 1186.60 | 1260.83 | 46.91 |

nP, number of estimable parameters
